# A classification of diabetic foot infections using ICD-9-CM codes: application to a large computerized medical database

**DOI:** 10.1186/1472-6963-10-192

**Published:** 2010-07-06

**Authors:** Benjamin G Fincke, Donald R Miller, Robin Turpin

**Affiliations:** 1 Center for Health Quality Outcomes and Economic Research (CHQOER), Bedford VA Medical Center, 200 Springs Road, Bedford, MA 01730 USA; 2 Boston University School of Public Health, Department of Health Policy and Management, 715 Albany Street, Boston, MA 02118 USA; 3 Merck & Co., One Merck Drive, Whitehouse Station, NJ 08889-0100 USA; 4 Department of Health Policy, Jefferson Medical College, 1015 Walnut Street, Philadelphia, PA 19107 USA

## Abstract

**Background:**

Diabetic foot infections are common, serious, and varied. Diagnostic and treatment strategies are correspondingly diverse. It is unclear how patients are managed in actual practice and how outcomes might be improved. Clarification will require study of large numbers of patients, such as are available in medical databases. We have developed and evaluated a system for identifying and classifying diabetic foot infections that can be used for this purpose.

**Methods:**

We used the (VA) Diabetes Epidemiology Cohorts (DEpiC) database to conduct a retrospective observational study of patients with diabetic foot infections. DEpiC contains computerized VA and Medicare patient-level data for patients with diabetes since 1998. We determined which ICD-9-CM codes served to identify patients with different types of diabetic foot infections and ranked them in declining order of severity: Gangrene, Osteomyelitis, Ulcer, Foot cellulitis/abscess, Toe cellulitis/abscess, Paronychia. We evaluated our classification by examining its relationship to patient characteristics, diagnostic procedures, treatments given, and medical outcomes.

**Results:**

There were 61,007 patients with foot infections, of which 42,063 were classifiable into one of our predefined groups. The different types of infection were related to expected patient characteristics, diagnostic procedures, treatments, and outcomes. Our severity ranking showed a monotonic relationship to hospital length of stay, amputation rate, transition to long-term care, and mortality.

**Conclusions:**

We have developed a classification system for patients with diabetic foot infections that is expressly designed for use with large, computerized, ICD-9-CM coded administrative medical databases. It provides a framework that can be used to conduct observational studies of large numbers of patients in order to examine treatment variation and patient outcomes, including the effect of new management strategies, implementation of practice guidelines, and quality improvement initiatives.

## Background

Diabetic foot infections are common and serious. They are diverse, and can range from cellulitis of a toe to gangrene of the foot. Diagnostic and treatment strategies are correspondingly varied. Thus, a very large study would be needed to evaluate how these infections are managed in actual practice and how outcomes might be altered by different treatment strategies. The use of computerized medical databases could address this problem. Such databases often include very large numbers of patients and are an important and increasing source of information for use in medical research. First, however, applicable methods are needed to identify and categorize patients with diabetic foot infections.

In this paper, we report the construction of a classification system for diabetic foot infections that is unique, in that it is expressly designed for use with ICD-9-CM coded administrative data and is derived from a very large number of patients. To our knowledge, no similar system has been described. We have applied it to a database that contains all diabetic patients in the Veterans Health Administration (VHA), and have found support for our classification in its relationship to patient characteristics, treatments received, and outcomes, including rehospitalization, amputation, transition to long-term care, and death. We developed it in preparation for a study of practice variation in antibiotic use, but it is readily applicable to other investigations. It provides a framework that can be used to conduct observational studies of large numbers of patients in order to examine treatment variation and patient outcomes, including the effect of new management strategies, implementation of practice guidelines, and quality improvement initiatives.

## Methods

### The Study Population and Source Data

This retrospective observational study was approved by the Institutional Review Board (IRB) of the Edith Nourse Rogers VA Medical Center in Bedford, Massachusetts. It was conducted in the population of diabetic patients receiving care from the Veterans Health Administration (VA) from 1998 through 2004. Our data came from the national VA Diabetes Epidemiology Cohorts (DEpiC), a linked, computerized research database that serves as a registry of virtually all VHA patients with diabetes. It contains patient-level data on medical visits, pharmacy and laboratory data, with diagnoses and procedures for VA and non-VA care (from Medicare claims data)[1].

### Identification of ICD-9 codes for diabetic foot infections

First we looked up alphanumeric diagnoses indicative of diabetic foot infections in the "index to diseases and injuries" of the ICD-9-CM coding manual. This index serves to "map" various alphanumeric diagnoses to their ICD-9-CM codes[2]. Then we reviewed the formal definitions of these ICD-9-CM codes to confirm that they did indeed indicate foot infection. We also examined the definitions of related codes sharing the same initial three-digit root. In addition, we used ICD-9 procedure codes and CPT-4 codes to identify patients with amputation (see appendix for codes). We then examined all the ICD-9-CM codes that had been assigned to these patients in the 90 days before the amputation code appeared in the database. We expected that codes for foot infection would appear, because foot infections are a frequent cause of amputation. Finally, we classified the resulting ICD-9-CM codes for foot infection into two groups, specific and moderately specific.

*Specific codes *for foot infection included all codes for osteomyelitis, cellulitis/abscess, paronychia, ulcer, and gangrene. We included the general codes for foot ulcers because 58%-68% of such ulcers are associated with active infection[3,4]. Likewise, we included the general codes for gangrene even though they do not specify whether it is infectious in cause, because infectious gangrene is such an important and serious condition. We also included the code for gas gangrene (which is always infectious). The codes for osteomyelitis, cellulitis/abscess, paronychia, ulcer, and gangrene all have subcodes that permit definite or highly presumptive localization to the foot. The exception is the code for gas gangrene, but on subsequent analyses patients with this code did not differ significantly in characteristics or outcomes from the other patients with gangrene.

*Moderately specific codes *were those for open wounds of the foot and infections of the leg (which we included to be comprehensive because the latter can sometimes arise by extension from the foot).

We were conservative in our choice of codes and eliminated those that either did not indicate the location of infection or allow the location to be confidently inferred. We did this to maximize the specificity of our codes for diabetic foot infections. Examples of codes that we did not include are 681.9, "Infection or abscess of unspecified digit," and 707.8, "Chronic ulcer of other unspecified sites." See the appendix for our final list of ICD-9-CM codes.

### Use of ICD-9-CM codes to classify diabetic foot infections

**Figure 1:**
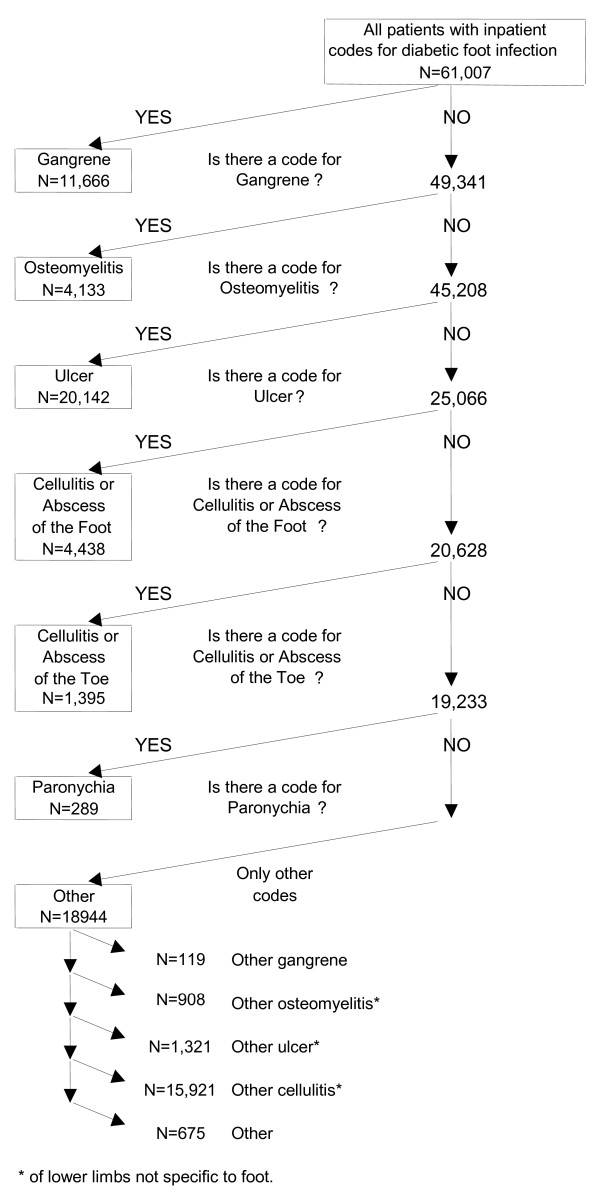
**VA patients with foot infections: classification to group**. * Of lower limbs, not specific to foot

Patients may have coexisting foot infections of different types. This raises the question of how they should be classified. We reasoned that the most severe of the infections would determine the strength of association with clinical outcomes, such as length of hospital stay, amputation rate, transition to long-term care, and mortality. Therefore, we ranked the infections in a presumptive order of severity and assigned the infection to the most severe category for which they had an ICD-9-CM code. Our presumptive order was Gangrene > Osteomyelitis > Foot ulcer > Cellulitis/abscess of foot > Cellulitis/abscess of toe > Paronychia. This was based on clinical judgment, but corresponds in part to Wagner's classification system for diabetic foot ulcers, which ranks ulcers with gangrene > ulcers with osteomyelitis, > ulcers alone[5,6]. To improve the homogeneity of the groups, we eliminated from each those patients who had only moderately specific codes for more severe types of infection. Figure 1 shows the flow of patients through this process, the numbers that were classified into each group, and the types of infections among those that remained unclassified.

We assumed that patients were under treatment for diabetic foot infection while in the hospital if, during that hospitalization, they were assigned an ICD-9-CM code for any of the foregoing types of diabetic foot infection.

### Characteristics and treatment of patients with diabetic foot infections

We described patients in terms of demographics, complications of diabetes, and co-existing medical conditions. Patient demographics were obtained from file entries for inpatient stays and outpatient visits spanning the years of available data (1987-2004). Complications of diabetes and other co-existing medical conditions were identified from ICD-9-CM codes in inpatient and outpatient records in the two-year period prior to the first hospitalization in our database that had an ICD-9-CM code for foot infection. Codes used were determined by VA clinicians using published evaluations whenever available. Cerebrovascular disease was identified with an algorithm evaluated in VA with cases confirmed by chart review and found to have high sensitivity (92%), albeit with moderate specificity (40%)[7,8]. On the other hand, the chronic kidney disease codes have low sensitivity (20%-42%) but high specificity (93%-99%) when compared with laboratory measures[9]. The codes for myocardial infarction also have high specificity[10].

#### Treatment during first identified hospitalization for foot infection

We listed all ICD-9-CM diagnosis and ICD-9 procedure codes that occurred for 1% or more of inpatients with foot infection. We eliminated those that were not surgical in nature, did not carry anaesthetic risk or that were not needed for DRG assignment, because such procedures are not required to be coded according to current guidelines. For amputations, we grouped all amputation codes and report the number of patients with any such code. (See the appendix for specific codes.)

### Outcomes of patients with diabetic foot infections

#### Rehospitalization for foot infection

Using ICD-9-CM codes and our classification system, we determined: 1) The proportion of patients who were rehospitalized for any type of foot infection; 2) The type of foot infection that was present; and, 3) The number of days between admissions.

#### Long-term outcomes

Long-term outcomes were amputation rate, transition to long-term care, and death. We included long-term care because serious disease of the lower extremity might result in impaired ambulation and a need for nursing home care in older patients--a phenomenon that has been observed in patients who have had surgery for hip fracture[11]. We included death because of the high frequency of co-morbid vascular disease, which is a predisposing factor for serious foot infections.

Subsequent amputations were identified by ICD-9 procedure codes from inpatient records. We also identified past amputation using the ICD-9 procedure codes along with ICD-9-CM diagnosis and CPT-4 procedure codes for post-amputation and prosthesis care; we used this broader definition because it is more sensitive in identifying amputations, including those that may have occurred outside of the VA[12,13].

Long-term care was identified from outpatient, inpatient, extended care, and fee basis files. The first three types of files provide information on VA long-term care. The last type provides records of community-based non-VA long-term care charged to the VA. Unfortunately, we do not have information on other community-based non-VA long-term care. Long-term care includes both inpatient (i.e. nursing home) and outpatient (home health and/or skilled nursing) care.

Deaths were identified from DEpiC, which assigns death based on an algorithm using information from the VA Beneficiary Identification and Records Locator Subsystem (BIRLS) file, VA inpatient records, the Social Security Death Index, and Medicare records. The combination of these records has a sensitivity of 98%[14].

### Statistical analyses

We used the Cochrane-Armitage test of trend to determine whether there was a statistically significant graded relationship between our severity ranking and clinical outcomes. We used a Bonferroni adjustment for multiple comparisons applied to an alpha of 0.05[15].

## Results

### Counts and classification of patients with diabetic foot infections

We identified 61,007 patients with one or more specific or moderately-specific codes for diabetic foot infection in inpatient records. Of these, 42,063 (68.9%) had specific codes that were classifiable into one of our predefined groups. Thirty-three percent of all patients had ulcer, 19.1% gangrene, 7.3% cellulitis or abscess of the foot, 6.8% osteomyelitis, 2.3% cellulitis or abscess of the toe, and 0.5% paronychia. As shown in figure 1, we classified the remaining 18,944 patients (31.1%) as having various "other" types of infection. This "other" category consisted almost entirely of patients with ICD-9-CM codes for infection of the leg but not specifically of the foot. For example, 84% of them had codes for cellulitis of the leg. They are included in our sample because infection of the foot can extend into the leg but they are not included in the evaluation of our classification system.

### Proportions of patients with co-existing infections

The types and proportions of co-infections are shown in Table 1. By definition, patients with a given infection cannot have any co-existing infections that are more severe, but may have those that are less severe.

**Table 1:** Coexisting Infections in Patients hospitalized for Diabetic Foot Infections

	Group					
	
						
Coexisting infections	Gangrene	Osteomyelitis	Ulcer	Foot Cellulitis or Abscess	Toe Cellulitis or Abscess	Paronychia
	
	11,666	4,133	20,142	4,438	1,395	289
						
**Gangrene**	-	-	-	-	-	-
						
**Osteomyelitis**	< 1%	-	-	-	-	-
						
**Ulcer**	17.2%	25.0%	-	-	-	-
						
**Foot cellulitis/abscess**	16.0%	23.5%	17.6%	-	-	-
						
**Toe cellulitis/abscess**	< 1%	11.6%	< 1%	< 1%	-	-
						
**Paronychia**	< 1%	< 1%	< 1%	< 1%	< 1%	-

### Demographics, diabetic complications, and other co-morbidities of patients with different types of infection

The demographic characteristics and diabetic complications of patients with different types of foot infection are shown in Table 2. African-Americans tended to have more severe infections. The exception to this was in patients with paronychia. Because of its mild nature, patients with this condition would almost certainly be admitted to hospital for other reasons. This affects their demographics and other factors, such as length of stay, hospital procedures, and co-morbid conditions. Patients with gangrene were older than those with other types of infection.

**Table 2:** Demographics and Diabetic Complications of Patients with Hospitalization for Diabetic Foot Infection

	Group							
	
	Any	Gangrene	Osteomyelitis	Ulcer	Foot Cellulites or Abscess	Toe Cellulitis or Abscess	Paronychia	Other
								
	**61,007**	**11,666**	**4,133**	**20,142**	**4,438**	**1,395**	**289**	**18,944**

								

Male	98.5%	99.1%	98.9%	98.6%	98.4%	98.4%	96.9%	98.0%

Mean Age	68.1	70.0	66.3	68.7	66.9	67.3	66.7	66.9

Standard deviation	11.0	10.2	11.0	10.9	11.4	11.4	12.6	11.3

								

White	76.3%	68.0%	75.1%	77.9%	76.6%	81.1%	76.6%	79.7%

African-American	16.0%	***23.1%***	***17.5%***	***15.7%***	***14.3%***	***12.0%***	17.4%	12.1%

Other race	7.7%	8.9%	7.4%	6.4%	9.1%	6.9%	6.0%	8.2%

Unknown	3.5%	3.1%	6.2%	3.1%	2.9%	3.3%	2.4%	3.7%

								

Peripheral vascular disease	66.9%	***77.2%***	71.5%	73.8%	58.4%	64.6%	55.7%	54.6%

Peripheral neuropathy	53.4%	55.6%	***62.7%***	***59.9%***	55.5%	58.1%	49.5%	42.5%

Diabetic eye disease	43.2%	48.9%	48.7%	47.3%	41.4%	46.5%	34.3%	34.2%

Cerebrovascular diseases	24.3%	29.2%	***19.2%***	26.1%	21.2%	24.7%	27.7%	21.0%

Myocardial infarction	22.5%	24.0%	***17.0%***	22.7%	20.5%	22.2%	23.5%	23.0%

Renal diseases	20.6%	26.2%	16.9%	22.1%	17.0%	18.5%	19.4%	17.5%

Text in large, bold, italic font shows findings that correspond to clinical expectation (see discussion).

Table 2 also shows that the prevalence of diabetic complications varies with our classification of diabetic foot infections. Patients with gangrene have the highest prevalence of peripheral vascular disease while patients with the mildest infection, paronychia, have the lowest prevalence of peripheral vascular disease and also diabetic eye disease. Patients with osteomyelitis and ulcer have the highest prevalence of neuropathy and the lowest prevalence of cerebrovascular disease and myocardial infarction.

We also examined the prevalence of 33 additional physical co-morbidities and 7 mental co-morbidities (data not shown). It was apparent that diabetic patients with foot infections have many additional serious problems: for example, hypertension (83.2%), obstructive chronic bronchitis (48.4%), congestive heart failure (39.4%), depression (37.8%) and cancer (33.8%).

### Length of hospital stay and treatments received

Table 3 summarizes length of stay and shows the proportion of patients with various treatments for each of our patient groups. There are a number of trends that correspond to the severity ranking embedded in our classification system. Most notably, the median length of stay decreases progressively from gangrene to cellulitis of the toe. Trends of increasing treatment with increasing severity are also present for any current amputation and any past amputation. When the trends are not entirely monotonic, the deviation is slight and occurs at the milder end of our severity ranking.

**Table 3:** Length of hospital stay and procedures of patients with hospitalization for diabetic foot infection

	Group						
	
	Gangrene	Osteomyelitis	Ulcer	Foot Cellulitis or Abscess	Toe Cellulitis or Abscess	Paronychia	Other
	**11,666**	**4,133**	**20,142**	**4,438**	**1,395**	**289**	**18,944**

**Length of stay (days)**							

Median	**14**	**10**	**7**	**6**	**5**	6	6

Mean(SD)	31.1 (61.6)	28.5 (62.5)	20.0 (62.5)	12.6 (34.9)	15.2 (86.6)	27.8 (96.5)	13.9 (44.4)

							

**Diagnostic studies**							

Arteriogram leg	***12.7%***	4.1%	5.8%	2.4%	3.3%	2.1%	1.6%

Aortogram	***7.8%***	2.6%	3.7%	1.6%	2.5%	< 1%	1.2%

							

**Treatments**							

Any current amputation	**1.9%**	**1.5%**	**0.3%**	**0.3%**	**0.7%**	**0.0%**	0.2%

Any past amputation	**11.0%**	**5.7%**	**4.2%**	**2.0%**	**2.0%**	**1.4%**	1.7%

Excisional debridement	3.5%	***5.4%***	3.8%	2.0%	2.1%	1.4%	1.0%

Skin/subcutaneous I & D	1.2%	2.5%	1.6%	***3.9%***	***3.8%***	2.8%	3.0%

							

**Outcomes at 1 year**							

Amputations	**18.8%**	**10.0%**	**7.7%**	**4.2%**	**5.4%**	**1.7%**	1.8%

Long-term care	**16.5%**	**14.6%**	**11.1%**	**10.5%**	**8.2%**	**7.9%**	8.6%

Death	**24.4%**	**12.5%**	**19.3%**	**11.5%**	**12.0%**	**14.9%**	13.2%

Rows with large bold font show statistically significant trends after correction for multiple comparisons. Cells with large bold italic font show findings that correspond to clinical expectation (as considered in discussion)

As expected, patients with different types of infection are identified as having different types of procedures. Those with cellulitis or abscess of the foot or toe most commonly have incision and drainage, while those with osteomyelitis most commonly have excisional debridement. Patients with gangrene undergo vascular radiographic studies and have amputation more frequently than others.

The table also shows that patients with paronychia have a length of stay disproportionately long for such a mild infection. They also have a more than two-fold increase in the prevalence of alcohol addiction and psychiatric treatment compared to the other patient groups (data for the latter two not shown). These findings are concordant with the likelihood that paronychial infection developed while the patient was in hospital for other reasons.

### Outcomes post-discharge

#### Rehospitalization for foot infection

A total of 24,297 patients (39.8%) had readmission for any type of foot infection over the 6-year time span of our database.

Patients initially hospitalized with the most severe types of infection were most often readmitted for the same problem (28% of those with gangrene, 25% of those with ulcer, 17% of those with osteomyelitis). Those initially hospitalized with milder types of infection were more commonly readmitted with more severe infections, particularly ulcer (cellulitis/abscess of the foot 12%, cellulitis/abscess of the toe 11%, paronychia 6%). The shortest time to readmission was for recurrence of the same type of infection for all types of initial infection other than ulcer. Ulcer patients returned earliest for gangrene, but next earliest for ulcer itself.

#### Rates of amputation, transition to long-term care, and death

Table 3 shows the proportions of each type of infection with amputation, transition to long-term care, and death in the year after discharge.

Overall, eight thousand and twenty-two patients (13.1%) had new ICD-9-CM codes for lower leg amputation at any time after their first hospitalization. Of these, 7.8% had an amputation within one year, with more than half (4.8%) occurring within 90 days. Amputation rates declined progressively with declining severity of initial infection, the only exception being patients with toe cellulitis/abscess (p < 0.001).

We examined transition to long-term care in the 47,582 patients (78.0%) that had no prior long-term care. Of these, 8.7% had subsequent inpatient (i.e. nursing home) long-term care within a year and for more than half of them (5.1%) this occurred within 90 days. As with amputation, transition to long-term care showed a progressive decline with decreasing severity of the initial infection (p < 0.001).

A total of 21,074 of the 61,007 patients (34.5%) with diabetic foot infection died after discharge. Roughly half this total (17% of patients) died within one year of hospital discharge and roughly half of those patients (8.8%) died within 90 days. Those with gangrene had the greatest risk. Though the trend in mortality in relation to our severity ranking is statistically significant, it is mostly driven by the very high mortality in patients with gangrene.

## Discussion

A number of classification systems for diabetic foot infections have been developed, but none of them was designed for use with ICD-9-CM coded administrative data[6,16-22]. In addition, those that consider infection do so in the context of a co-existing diabetic foot ulcer. This is understandable, since foot ulcers are by far the most common antecedent of infection[23]. In contrast, we have designed our classification system expressly for use with ICD-9-CM coded data, and have included infections that may sometimes occur in the absence of ulcer.

Our classification is made up of six mutually exclusive types of infection. This is obligatory for comparing one type of infection to another in relation to outcomes, utilization of resources, and other matters of interest. Substantial numbers of patients, however, have more than one kind of infection. We have addressed this by creating a hierarchy of severity. An important question is whether our hierarchy is sound.

A number of findings support the validity of our severity ranking. First, we have found that African-Americans tended to have more severe infections, which agrees with the known higher frequency of amputation in non-whites[24]. Second, with minor exceptions, we found a statistically significant monotonic relationship of our scale to hospital length-of-stay, past amputation, current amputation, subsequent amputation, and transition to long-term care. These findings are consonant with those of Pittet, in a study of foot ulcers. He found failure rates of treatment in 93% in patients who had an ulcer accompanied by gangrene, 30% when accompanied by osteomyelitis, and 19% in the remainder[5]. It is to be expected that infections that are more difficult to treat will increase the duration of hospitalization, raise the rates of amputation, and result in a greater need for long-term care.

These findings corroborate our severity ranking. They also confirm our ability to categorize infections correctly, because it would not be possible to create a workable ranking otherwise. There is additional support in our finding that the shortest time to readmission was for the same type of infection, with longer intervals for new types of infection. Further evidence derives from the relationship of our categories of infection to expected patient characteristics and treatments. Thus, gangrene, which is related to vascular insufficiency,[25] has the highest prevalence of peripheral vascular disease as well as a higher frequency of vascular radiographic studies. Likewise, both ulcer and osteomyelitis have a higher frequency of peripheral neuropathy, with which they are known to be associated[23]. Osteomyelitis complicates severe neuropathic ulcers, which can occur independently of macrovascular disease[26]. As expected, patients with osteomyelitis are more often are treated with excisional debridement, which is particularly indicated for removal of infected bone[23]. Cellulitis/abscess of the foot and toe were treated more often than other infections with incision and drainage.

The foregoing findings support the validity of our diagnostic categories and our severity ranking. The differences that we have found between types of infection, however, are sometimes small. There are a number of possible reasons for this. First, a significant number of our patients have co-existing infections, which would tend to blur distinctions among them. Second, if a patient received an ICD-9-CM code for foot infection during a hospitalization we have presumed that the infection was under treatment, but that was not necessarily the case. Third, there is the likelihood of undercoding and miscoding of diagnoses and treatments. This is a recognized problem in dealing with data derived from administrative records[27-29]. There is evidence that it has occurred in this study as well. For example, osteomyelitis had a significantly higher rate of excisional debridement compared to other infections, but the absolute increase in percentage was only 1.5. Undercoding occurs much less often for major co-morbidities, treatments and outcomes, however[30]. The percentage of ulcer patients in our study with amputation or death, for example, is comparable to the findings in other investigations[31,32].

Another limitation is that we do not have measures of severity within each category of infection. Other studies have demonstrated that gradations of severity in foot ulcers have an effect on outcomes[22,33,34]. Our classification can address this, however, if patients with ulcers are stratified according to whether they also have gangrene, osteomyelitis, or neither. This approach approximates the severity ranking created by Wagner[6]. Most such rankings, though, use clinical data that are not often present in computerized databases, such as size, depth, and signs of inflammation. This may change in the future. The data available in such databases are increasing and one database already exists that contains information about ulcer size and grade[35]. Such information can be incorporated into our classification as it becomes available.

We also presumed gangrene and foot ulcers to be infected, but this is not always the case. Where this is a concern, it can be addressed by restricting analyses to patients where infection is definite, as is the case when there is gas gangrene, osteomyelitis, cellulitis, or abscess.

Last, we have only applied statistical tests of significance to our trend analyses, because we considered them in designing the study as a hypothetical way to test the hierarchical order of infections in our classification system. The remaining observations are descriptive so we do not present results of statistical tests.

Despite the foregoing limitations, we have succeeded in showing that useful information can still be obtained, because we can distinguish different patterns of care and outcomes related to the particular infections that we have identified. In addition, the picture of diabetic foot infections that has emerged is consistent with that derived from investigations of foot ulcers, which are the most common predecessor of foot infection[23]. The findings in this study that correspond to the observations of others include: 1) A tendency for infections to be both recurrent and progressive, which is evident in our data on rehospitalization, and which fits with the known tendency of foot ulcers to persist and advance over time;[31,36] 2) The relationships of gangrene with peripheral vascular disease[25] and of peripheral neuropathy with foot ulcers and osteomyelitis;[23] 3) Progressively higher amputation rates in relation to ulcer, osteomyelitis, and gangrene;[5,33] and 4) The association of our infections with high short-term mortality, probably related to the high prevalence of co-existing macrovascular disease and other co-morbidities[32]. Beyond this, we have also contributed useful new information on readmissions, as well as on rates of amputation, transition to long-term care and death as they relate to patients with different categories of infection.

We have found no comparable studies reported in the literature. Other investigators have used ICD-9-CM codes as a case-finding strategy to choose medical records for manual review[37]. In one published study, chart diagnoses were compared to ICD-9-CM codes and were found to have a high sensitivity and specificity (0.99 and 0.93, respectively)[38]. A more recent study from Australia, however, showed much lower sensitivity[39]. This divergence may reflect differences in coding practices. This carries the implication that our classification system needs to be confirmed in other systems of care, and, depending on the findings, it is possible that our choice of ICD-9-CM codes might need to be modified.

## Conclusions

We have developed a classification system for patients with diabetic foot infections for use with large, computerized, administrative medical databases. It is broadly applicable, because it is based upon ICD-9-CM codes that are in widespread use. If further validated in other settings, it will provide a framework that can be used to conduct observational studies of large numbers of patients in order to examine factors that influence patient outcomes, such as new treatments, implementation of practice guidelines, quality of care, and practice variation.

## Competing interests

The authors declare that they have no competing interests.

## Authors' contributions

All three authors contributed to the conception, design, drafting, interpretation of findings, and final approval of the manuscript. DRM acquired the data and oversaw the analysis.

## Appendix

### Codes for amputation involving the lower extremity

*Current amputations (ICD-9 procedure codes):*84.11-17

*Past amputations (ICD-9-CM codes): *V49.71-77; V52.1 (CPT-4 codes): 27888, 28800, 28801, 28802, 27803, 28804, 28805, 27290, 27598, 27880, 27881, 27882, 27884, 27885, 27886, 27590, 27591, 27592, 27290, 27291, 27292, 27293, 27294, 27295, 27594, 27595, 27596, 26910, 28810, 28811, 28812, 28813, 28814, 28815, 28816, 28817, 28818, 28819, 28820, 28821, 28822, 28823, 28824, 28825

### Final set of ICD-9-CM codes for foot infection

Gangrene

040.0Gas Gangrene

440.24Atherosclerosis of the extremities with gangrene

785.4Gangrene but only if any one of the following is also present:

250.7 Diabetes with peripheral circulatory disorders

440.2 Atherosclerosis of native arteries of the extremities

Any condition classifiable to 440.21, 440.22, and 440.23

Osteomyelitis

730.07 Acute osteomyelitis of ankle and foot

730.17 Chronic osteomyelitis of ankle and foot

730.27 Unspecified osteomyelitis of ankle and foot

730.97 Unspecified infection of bone of ankle and foot

Ulcer

440.23 Atherosclerosis of the extremities with ulceration

707.14 Ulcer of heel and mid foot

707.15 Ulcer of other part of foot

707.1 Ulcer of lower limbs

Cellulitis or abscess of foot

680.7 Carbuncle and furuncle of foot, heel, toe

682.7 Cellulitis and abscess of foot, except toes

Cellulitis or abscess of toe

681.1 Cellulitis and abscess of toe

681.10 Cellulitis, toe nos

Paronychia

681.11 Onychia and paronychia of toe

### ICD-9-CM codes for complications of diabetes

*Peripheral vascular disease: *250.7, 440, 443.8, 443.9, 785.4, 997.2

*Peripheral neuropathy: *250.6, 357.2

*Diabetic eye disease: *250.5, 362.0, 379.23

*Cerebrovascular disease: *435 or [primary diagnosis = 430-432, 434, 436] OR

[V57.xx (rehab) AND secondary diagnosis = 342 (hemiparesis), 430-438] OR

[primary diagnosis = 433, 435 AND secondary diagnosis = 342, 430-432, 434, 436]

*Myocardial infarction: *410, 411.0, 427.5

*Renal disease: *585, 586, 996.73, 996.81, V42.0, V45.1

### CPT-4 codes for treatments

*Angiography, extremity: *75710, 75716

*Aortography, abdominal, plus bilateral iliofemoral lower extremity: *75630

*Aortography, abdominal or thoracic: *75600, 75605, 75625

*Debride tissue/muscle/bone: *11044

*Incision and drainage of abscess: *10060, 10061, 20000, 20005

## Pre-publication history

The pre-publication history for this paper can be accessed here:

http://www.biomedcentral.com/1472-6963/10/192/prepub
